# Supplementary material for: Prognostic impact of the combination of serum transaminase and alkaline phosphatase determined in the emergency room in patients with ST-segment elevation myocardial infarction undergoing primary percutaneous coronary intervention
Source: PLoS One. 2020 May 22;15(5):e0233286. doi: 10.1371/journal.pone.0233286 (PMC7244093; doi:10.1371/journal.pone.0233286)
Supplement: S1 Table — (PDF) [file pone.0233286.s001.pdf]

S1 Table. Discharge medication data among MI survivors

	All (n=1139)	HLI† (-) & low ALP (n=465)	HLI (-) & high ALP (n=463)	HLI (+) & low ALP (n=106)	HLI (+) & high ALP (n=105)	p value
Aspirin	1134 (99.6)	463 (99.6)	462 (99.8)	104 (98.1)	105 (100.0)	0.108
P2Y12 inhibitor	1127 (98.9)	461 (99.1)	459 (99.1)	104 (98.1)	103 (98.1)	0.623
Beta-blocker	1060 (93.3)	436 (93.8)	429 (92.9)	96 (90.6)	99 (96.1)	0.410
ACEI or ARB	898 (78.9)	368 (79.1)	361 (78.1)	86 (81.1)	83 (79.0)	0.920
Statin	995 (87.4)	408 (87.7)	412 (89.0)	89 (84.0)	86 (81.9)	0.164

HLI, hypoxic liver injury; ALP, alkaline phosphatase; ACEI, angiotensin converting enzyme inhibitors; ARB, angiotensin II receptor blockers

†Hypoxic liver injury (HLI) was defined as an elevation of serum transaminase levels to more than twice the upper limit of normal.
