# Supplementary material for: Prognostic impact of the combination of serum transaminase and alkaline phosphatase determined in the emergency room in patients with ST-segment elevation myocardial infarction undergoing primary percutaneous coronary intervention
Source: PLoS One. 2020 May 22;15(5):e0233286. doi: 10.1371/journal.pone.0233286 (PMC7244093; doi:10.1371/journal.pone.0233286)
Supplement: S2 Table — (PDF) [file pone.0233286.s002.pdf]

S2 Table. Incidence of adverse clinical outcomes according to levels of serum ALT and ALP

	All (n=1176)	ALT (-)† & low ALP (n=551)	ALT (-) & high ALP (n=554)	ALT (+)† & low ALP (n=34)	ALT (+) & high ALP (n=36)	p value for trend
In-hospital death	37 (3.1)	13 (2.4)	16 (2.9)	2 (5.7)	6 (16.7)	<0.001
MACCE	142 (12.1)	49 (8.9)	76 (13.7)	7 (20.0)	10 (27.8)	<0.001
All-cause death	64 (5.4)	24 (4.4)	29 (5.2)	3 (8.6)	8 (22.2)	<0.001
Non-fatal myocardial infarction	36 (3.1)	9 (1.6)	25 (4.5)	2 (5.6)	0 (0)	0.107
Ischemia-driven revascularization	30 (2.6)	10 (1.8)	17 (3.1)	2 (5.7)	1(2.8)	0.154
Non-fatal stroke	12 (1.0)	6 (1.1)	5 (0.9)	0 (0)	1 (2.8)	0.824

ALT, alanine transaminase; ALP, alkaline phosphatase; MACCE, major adverse cardiac and cerebrovascular events

†ALT (-) and ALT (+) mean the patient group who had serum ALT level ≤ 80 U/L and > 80 U/L, respectively.
