# Supplementary material for: Prognostic impact of the combination of serum transaminase and alkaline phosphatase determined in the emergency room in patients with ST-segment elevation myocardial infarction undergoing primary percutaneous coronary intervention
Source: PLoS One. 2020 May 22;15(5):e0233286. doi: 10.1371/journal.pone.0233286 (PMC7244093; doi:10.1371/journal.pone.0233286)
Supplement: S1 Fig — †The Model 1 included age, sex, diabetes mellitus, hypertension, ejection fraction, Killip class, anterior myocardial infarction, symptom to balloon time (log 10) and peak creatine kinase-myocardial band isoenzyme (log 10). (PDF) [file pone.0233286.s003.pdf]

S1 Fig. Receiver operating curves for the predicted probabilities of selected risk models before (blue line) and after (red line) the addition of hypoxic liver injury (HLI) and alkaline phosphatase (ALP, >73 IU/L) status to the multivariate Model 1.

†The Model 1 included age, sex, diabetes mellitus, hypertension, ejection fraction, Killip class, anterior myocardial infarction, symptom to balloon time (log 10) and peak creatine kinase-myocardial band isoenzyme (log 10).

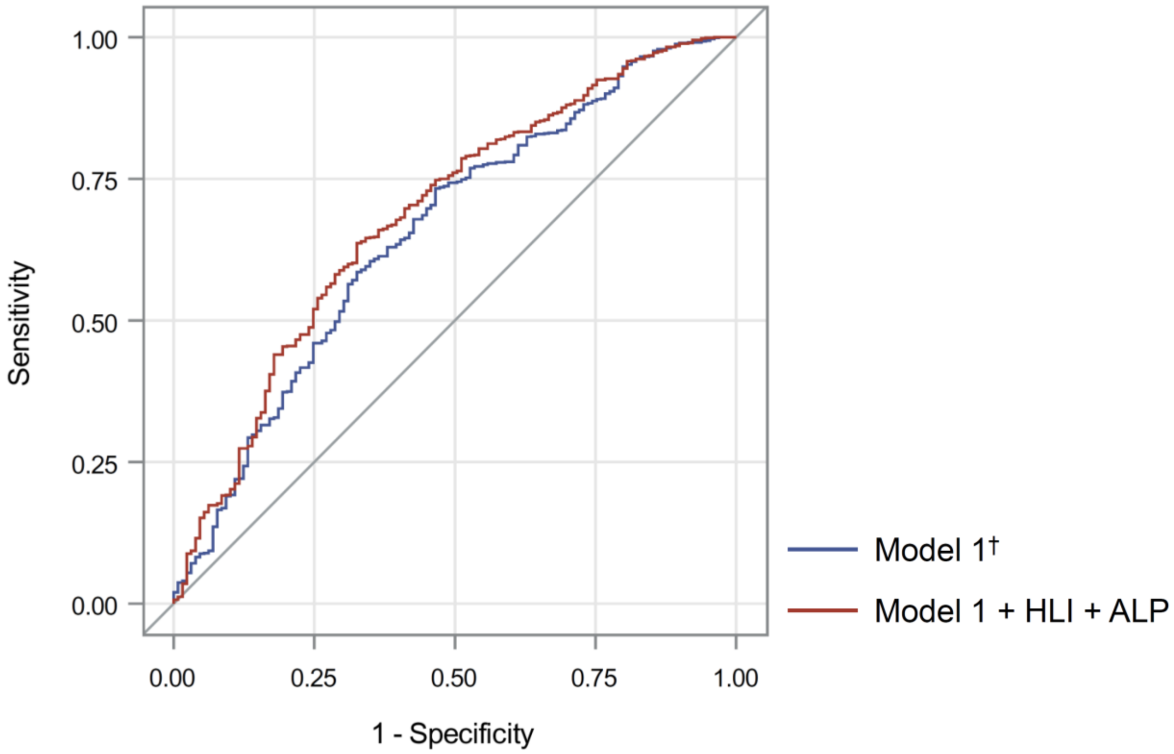

Multivariate model	C-Statistic	95% CI	p value
Model 1	0.6559	0.6038-0.7080	<0.001
Model 1 + HLI + ALP	0.6854	0.6350-0.7358	<0.001
Difference	0.0295	0.0015-0.0583	0.044
